# Hydrodynamic flow of non-Newtonian power-law fluid past a moving wedge or a stretching sheet: a unified computational approach

**DOI:** 10.1038/s41598-020-66106-6

**Published:** 2020-06-10

**Authors:** Ramesh B. Kudenatti, Noor-E- Misbah

**Affiliations:** Department of Mathematics, Bengaluru Central University, Central College Campus, Bengaluru, 560 001 India

**Keywords:** Applied mathematics, Computational science

## Abstract

A unified mathematical equation that combines two different boundary-layer flows of viscous and incompressible Ostwald-de Waele fluid is derived and studied. The motion of the mainstream and the wedge is approximated in the power-law manner, i.e, in terms of the power of the distance from the leading boundary-layer edge. It is also considered that the wedge can move in the same and opposite direction to that of the mainstream. The governing partial differential equations are transformed into the nonlinear ordinary differential equation using a new set of similarity variables. This transformed equation subjected to the boundary conditions describing the flow is then solved using the Chebyshev collocation method. Further, these numerical results are then validated by determining the flow behaviour at far-field by performing asymptotics. The velocity ratio parameter effectively captures and distinguishes two boundary-layer flows. The boundary layer thickness for shear-thinning fluid is thinner compared to corresponding parameters for shear-thickening fluids and is markedly separated by the Newtonian fluid. Further, the boundary-layer flow of the non-Newtonian fluid predicts an infinite viscosity for shear-thinning fluid quite away from the surface. The hydrodynamics of the obtained results is explained thoroughly.

## Introduction

Boundary-layer flow over a surface is one of the fundamental fluid dynamical problems in understanding the aerodynamical properties such as the wall shear stress, drag, etc. For example for the fluid flow over a flat plate(Blasius flow), the wall shear stress is 0.336152. The same problem was then extended by Falkner-Skan by considering the flow over a wedge and gave a numerical solution for different pressure gradients. The same Blasius problem later modified by Sakiadis^1^ in which the flat plate is allowed to move or stretch along its own axis with sufficiently large Reynolds number in a still fluid, the calculated wall shear stress is 0.4696. In the above studies, either the surface or the fluid is assumed to be at rest. However, there are industrial applications in which both surface and fluid are moving. This case apparently is significant in aerodynamics. This situation is extensively studied in the literature (Klemp & Acrivos^2^; Riley & Weidman^3^; Sachdev *et al*.^4^; Kudenatti *et al*.^5,6^, Fang^7,8^) in different aspects. Riley & Weidman^3^ have shown that there are multiple solutions to the problem when the wedge surface is considered to move in the same or opposite direction to that of mainstream flows. On the other hand, there are also numerous papers that considered the Sakiadis problem and the same is extended in different aspects, for example, the boundary-layer flow due to a stretching sheet in a still fluid finds various applications in an extrusion of plastic sheets, paper production, polymer sheet extrusion, metal spinning, glass blowing, etc. The boundary-layer flow in the presence of magnetic field (Andersson *et al*.^9^; Cortell^10^; Ishak *et al*.^11^; Prasad *et al*.^12^; Reddy *et al*.^13^;Turkyilmazoglu^14^; Sharma *et al*.^15^), porous medium (Cortell^16^; Mishra & Singh^17^; Singh *et al*.^18^), heat and mass transfer (Afzal^19^; Ishak *et al*.^20^; Cortell^21^; Hayat *et al*.^22^; Kudenatti *et al*.^23^; Kudenatti & Jyothi^24^), nanofluid (Khan & Pop^25^; Turkyilmazoglu^26^; Ziami *et al*.^27^ Othman *et al*.^28^; Dinarvand *et al*.^29^), micro-polar fluid (Ishak *et al*.^30^) etc.

The above mentioned hydrodynamic problems essentially show that the boundary-layer forms either due to the stretching of surface or forms over the moving (or at rest) wedge surface, and the resulting mathematical equations look similar but their description of motion is entirely different. Denoting $U(x)$ and ${U}_{w}(x)$ as the velocity of the mainstream and the wedge surface respectively, let $\varepsilon$ be the ratio of the wedge velocity to the mainstream velocity. The parameter $\varepsilon$ mathematically simplifies and unifies the above mentioned two boundary-layer flow problems for $\varepsilon =0$ and $\varepsilon =1$ (see section 2 for details). In other words, the mathematical equation for $\varepsilon =0$ that represents the boundary layer flow due to a stretching surface whose solution is substantially different from the boundary-layer flow over a constant wedge ($\varepsilon =1$). In this line of studies, Afzal^31^ derived a new type of boundary-layer flow problem by modifying the similarity transformations that produce $\varepsilon$ into it and solved numerically. Kudenatti^32^ revisited the same problem and solved it by uniformly valid convergent series solution in the range of $\varepsilon \in [0,0.5]$. Recently, Karkera *et al*.^33^ extended it by including the magnetic field and attempted the Haar wavelet-based numerical technique and compared their results with existing literature for various $\varepsilon$ values. In the present problem, a unified mathematical model is again derived for the hydrodynamic boundary-layer flow of a power-law fluid (Ostwald- De Waele) over a wedge, since the flow of non-Newtonian fluid has ample applications in chemical industries.

There are numerous applications of power-law fluids in various manufacturing industries. Sewage sludge, china clay, oil-water emulsion, cosmetics, paints, synthetic lubricants, biological fluids, jam, jellies, etc are some of the non-Newtonian fluids (Schowalter^34^; Bird *et al*.^35^, Andresson & Irgens^36^; Shenoy & Mashelkar^37^, etc). For these fluids, the viscosity or apparent viscosity essentially depends on the shearing rate. Acrivos *et al*.^38^ theoretically analyzed the power-law fluid flow and heat transfer over a surface considering various external surfaces. Mitschka & Ulbrecht^39^ and Andersson *et al*.^40^ have numerically presented the basic solutions for the shear-thickening and shear-thinning power-law fluids. However, the solutions shown by these authors did not match the mainstream properly. This issue is later fixed by Denier & Hewitt^41^ by modifying the similarity solutions of the boundary-layer flow for shear-thinning and shear-thickening fluids. Ishak *et al*.^42^ considered the boundary-layer flow of power-law fluid over a moving wedge for which the model shows non-unique solution structures which are similar to the Newtonian case. The linear stability of Griffith *et al*.^43^ shows that the stationary spiral instabilities observed experimentally can be exactly described by shear-thinning fluids. Griffiths^44^ has discussed the stability of shear-thinning boundary-layer flow of the power-law fluids in which the base flow is obtained using the similarity transformations of the Prandtl boundary layer equations for non-Newtonian fluid. He further discusses that the viscosity of the power-law fluid remains unbounded for shear-thinning fluid which is unphysical. Denier & Dabrowski^45^ have identified a non-unique solution to the shear-thickening and shear-thinning fluid flow in the boundary-layer. They further have shown asymptotically that the decaying of shear-thinning solutions into the mainstream is strongly algebraic.

In the present study, an attempt is made to predict the steady boundary-layer flow behaviour of a non-Newtonian power-law fluid over a stretching surface or a moving wedge whose speed is approximated in terms of the power of the distance from the leading edge of the boundary-layer. This work may be regarded as an extension of the flow problem encountered by Kudenatti^32^ to a class of non-Newtonian fluid known as Ostwald-de-Waele fluid. Since the resultant equation is highly nonlinear, we employ the Chebyshev collocation method(CCM) for the computation of the pertinent results. The CCM is a powerful technique to predict flow behaviour in the boundary-layer. There are very few papers that use CCM in the context of boundary-layer, however, for nonlinear or mild nonlinear see^46^.

Organization of the paper is as follows. We discuss the formulation of the problem in section 2 and the self-similar solutions are computed with suitable similarity transformations. The methodology applied to solve the flow problem is discussed in detail in section 3. To assess the nature of these obtained numerical solutions, we predict the flow behaviour of the system asymptotically from a large distance away from the origin of the boundary layer in section 4 known as far-field behaviour. Section 5 enunciate the results and the corresponding hydrodynamics is discussed in detail and a brief conclusion of the study is made in section 6.

## Formulation

**Figure 1:**
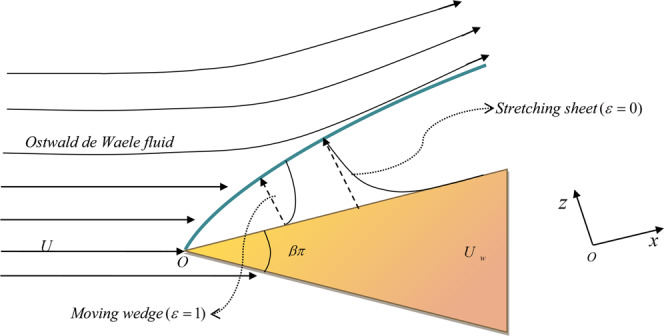
A schematic diagram representing boundary-layer formation in two distinct mathematical models under consideration.

We study the laminar flow of a steady, incompressible non-Newtonian power-law fluid over a semi-infinite moving wedge or due to a stretching sheet in a two-dimensional space. Let the streamwise coordinate along the wall be $x$ and normal to the wall be $z$ (refer Fig. 1 for detailed geometric representation). The flow is governed by the two-dimensional Navier-Stokes equation accompanied by continuity equation

1 $$\nabla \cdot v=0$$

2 $$\rho (v\cdot \nabla )v=-\,\nabla p+\nabla \cdot \tau $$

where ***v*** = $(u,v)$ is the velocity vector, $\rho$ is the density of the fluid, $p$ is the pressure, $\tau$ is the deviatoric stress tensor. The stress tensor for the power-law fluid is depicted by the relation

3 $$\tau =-\,\mu (\dot{{\boldsymbol{\gamma }}})\dot{{\boldsymbol{\gamma }}}$$

where $\dot{{\boldsymbol{\gamma }}}$ is the second invariant of the strain-rate tensor and the constitutive viscosity relation is described by the Ostwald-de Waele power-law model (Bird *et al*.^35^)

4 $$\mu =-\,{\mathscr{K}}|\dot{{\boldsymbol{\gamma }}}{|}^{m-1}\mathrm{}.$$

Here ${\mathscr{K}}$ and $m$ respectively denote the consistency index and the degree of non-Newtonian behavior of the fluid (Denier & Dabrowski^45^). The equations governing the model are non-dimensionalized with appropriate dimensionless variables

5 $$(x{\prime} ,z{\prime} )=\left(\frac{x}{L},\frac{z}{\delta L}\right),(u{\prime} ,v{\prime} )=\left(\frac{u}{U},\frac{v}{\delta U}\right),p{\prime} =\frac{p}{\rho {U}^{2}},$$

to obtain

6a $$\frac{\partial u{\prime} }{\partial x{\prime} }+\frac{\partial v{\prime} }{\partial z{\prime} }=0$$

6b $$u{\prime} \frac{\partial u{\prime} }{\partial x{\prime} }+v{\prime} \frac{\partial u{\prime} }{\partial z{\prime} }=-\,\frac{\partial p{\prime} }{\partial x{\prime} }+\frac{1}{Re}\left(\frac{\partial {\tau }_{x{\prime} x{\prime} }}{\partial x{\prime} }+\frac{\partial {\tau }_{z{\prime} x{\prime} }}{\partial z{\prime} }\right)$$

6c $$\delta \left(u{\prime} \frac{\partial v{\prime} }{\partial x{\prime} }+v{\prime} \frac{\partial v{\prime} }{\partial z{\prime} }\right)=-\,\frac{1}{\delta }\frac{\partial p{\prime} }{\partial z{\prime} }+\frac{1}{Re}\left(\frac{\partial {\tau }_{x{\prime} z{\prime} }}{\partial x{\prime} }+\frac{\partial {\tau }_{z{\prime} z{\prime} }}{\partial z{\prime} }\right)$$

where $L$, $U$ are appropriate scale factors for reference quantities to length and mainstream velocity respectively and $\delta$ denotes boundary-layer thickness. The non-Newtonian viscosity $\mu$ is referred to as an apparent viscosity ${\mu }_{app}$, wherein

7a $${\tau }_{ij}={\mu }_{app}\left(\frac{\partial {u}_{i}}{\partial {x}_{j}}+\frac{\partial {u}_{j}}{\partial {x}_{i}}\right)$$

7b $${\mu }_{app}={\mathscr{K}}{|2{\left(\frac{\partial u}{\partial x}\right)}^{2}+2{\left(\frac{\partial v}{\partial z}\right)}^{2}+{\left(\frac{\partial u}{\partial z}+\frac{\partial v}{\partial x}\right)}^{2}|}^{\frac{m-1}{2}}\mathrm{}.$$

From (7) in terms of (5) we have

8 $$({\tau }_{x{\prime} x{\prime} },{\tau }_{z{\prime} z{\prime} })=2\left(\frac{U}{L}\right){\mu }_{app}\left(\frac{\partial u{\prime} }{\partial x{\prime} },\frac{\partial v{\prime} }{\partial z{\prime} }\right),\,({\tau }_{z{\prime} x{\prime} },{\tau }_{x{\prime} z{\prime} })=\left(\frac{U}{L}\right){\mu }_{app}\left(\frac{1}{\delta }\frac{\partial u{\prime} }{\partial z{\prime} }+\delta \frac{\partial v{\prime} }{\partial x{\prime} }\right)\mathrm{}.$$

Further, the dimensionless apparent Reynolds number for the non-Newtonian fluid exhibits the form

9 $$Re=\frac{\rho {U}^{2-m}{L}^{m}}{{\mathscr{K}}}$$

which reduces to the Newtonian fluid when $m=1$. To this end, the two-dimensional boundary layer flow of non-Newtonian fluid is studied in which a single mathematical equation unifies two types of boundary-layer flows: over a moving wedge of an included angle $\pi \beta$ and due to nonlinear stretching of the surface. In the former case, for the flow of mainstream fluid $U=U(x)$ at high Reynolds number the viscosity effects are confined to a small region near the immediate vicinity of the wall, known as the boundary-layer region (Yuan^47^; Kudenatti^5^). In the latter situation, the boundary-layer forms in a still fluid ($U(x)=0$) due to stretching of the surface with large Reynolds number (Acheson^48^). These two flow problems have been mathematically combined into a single equation that is derived for the first time in the literature. Substituting (8) in (6) and making use of the fact that $Re$ is large and using the other boundary layer approximations and retrieving back to original variables, we get

10a $$\frac{\partial u}{\partial x}+\frac{\partial v}{\partial z}=0$$

10b $$u\frac{\partial u}{\partial x}+v\frac{\partial u}{\partial z}=-\,\frac{1}{\rho }\frac{dp}{dx}+\frac{{\mathscr{K}}}{\rho }\frac{\partial }{\partial z}\left({|\frac{\partial u}{\partial z}|}^{m}\right).$$

The conditions appropriately describing the flow are

11a $$u={U}_{w}(x),\,v=0\,{\rm{on}}\,z=0$$

11b $$u\to U(x)\,{\rm{as}}\,z\to \infty .$$

The boundary conditions (11) infer that the velocity of the fluid over the wedge surface asymptotically approaches the freestream far away from the wedge. Here $U(x)$ is the mainstream velocity and ${U}_{w}(x)$ is the velocity with which the wedge or stretching sheet moving in the boundary layer and approximating these velocities in a power-law manner

12 $$U(x)={U}_{\infty }{x}^{n},\,{U}_{w}(x)={U}_{0}{x}^{n}$$

where ${U}_{\infty },{U}_{0}$ and $n$ are arbitrary constants. The above forms exhibit a self-similar solutions in the boundary-layer in the Ostwald-de Waele fluid (Schowalter^34^). Further, from Bernoulli’s theorem for an incompressible flow of non-Newtonian fluid over a moving wedge, we have with $u=U$ on the edge of the boundary layer

13 $$-\,\frac{1}{\rho }\frac{dp}{dx}=\{\begin{array}{ll}0 & {\rm{for}}\,U(x)=0\,({\rm{a}}\,{\rm{still}}\,{\rm{fluid}})\\ U\frac{dU}{dx} & {\rm{for}}\,{\rm{moving}}\,{\rm{mainstream}}.\end{array}$$

Consequently, the momentum Eq. (10b) becomes, in general

14 $$u\frac{\partial u}{\partial x}+v\frac{\partial u}{\partial z}=U\frac{dU}{dx}+\frac{{\mathscr{K}}}{\rho }m{|\frac{\partial u}{\partial z}|}^{m-1}\frac{{\partial }^{2}u}{\partial {z}^{2}}.$$

For a still fluid we have $U(x)=0$ in (14) and otherwise (14) holds good for moving fluid. Accordingly, we define a single unified set of similarity transformations

15 $$\psi (x,z)=\frac{{U}_{r}(x)}{A{x}^{q}}\phi (\eta ),\,{\rm{with}}\,\eta =A{x}^{q}z$$

where *A*, *q* are unknowns to be determined and *U*_*r*_ is a suitable reference velocity defined as ${U}_{r}(x)=U(x)+{U}_{w}(x)=({U}_{\infty }+{U}_{0}){x}^{n}$ (Kudenatti^32^), $\psi (x,z)$ is the normalized stream function and $\eta$ is the similarity variable. Defining the stream function such that it satisfies continuity Eq. (10a) identically

16 $$(u,v)=\left(\frac{\partial \psi }{\partial z},-\,\frac{\partial \psi }{\partial x}\right)$$

with

17 $$A=\mathrm{(1}+\mathrm{(2}m-\mathrm{1)}n)({U}_{\infty }+{U}_{0}{)}^{2-m}\frac{\rho }{2{\mathscr{K}}}\,{\rm{and}}\,q=\frac{\mathrm{(2}-m)n-1}{m+1},$$

to obtain a highly nonlinear third-order differential equation

18 $$m|\phi {\prime\prime} {|}^{m-1}\phi {\prime} {\prime} {\prime} +\frac{2}{m+1}\phi \phi {\prime\prime} +\beta ({\varepsilon }^{2}-\phi {{\prime} }^{2})=0$$

where $\beta =\frac{2n}{1+(2m-1)n}$ is the generalized pressure gradient parameter, $\varepsilon =\frac{U(x)}{{U}_{r}(x)}=\frac{{U}_{\infty }}{{U}_{\infty }+{U}_{0}}$ is the velocity ratio parameter and the boundary conditions enclosing the system are

19 $$\phi (0)=0,\,\phi {\prime} (0)=1-\varepsilon \,{\rm{and}}\,\phi {\prime} (\infty )=\varepsilon .$$

We can enunciate the following cases depicted in Table 1 from (18) and (19) for different parameters affiliated with the system. From the table, it is clear that the governing system (18) and (19) unifies many physical models which are presented in the literature.

**Table 1:** Deduction of various cases of (18) and (19) to former investigatons.

Cases	*β*	*ε*	Investigator(s)
***m*** ** = 1 (Newtonian fluid)**			
1.	$\frac{2n}{n+1}$	1	Evans^55^; Liao^56^; Alizadeh *et al*.^57^
2.	1	1	Hiemenz^58^; Alizadeh *et al*.^57^
3.	0	1	Blasius flow
4.	$\frac{2n}{n+1}$	0	Afzal *et al*.^59^; Sachdev *et al*.^60^; Vajravelu & Cannon^61^; Kudenatti *et al*.^23^
5.	$\frac{4}{3}$	0	Kudenatti *et al*.^23^
6.	1	0	Crane^62^; Vajravelu & Cannon^61^;
7.	$\frac{2n}{n+1}$	*ε*	Afzal^31^; Kudenatti^32^ (analytic)
***m*** **≠ 1 (Power-law fluid)**			
8.	$\frac{2n}{(2m-1)n+1}$	1	Postelnicu & Pop^63^; Ishak *et al*.^42^
9.	0	1	Shashidhar *et al*.^13^ (MHD)
10.	$\frac{2}{3-m}$	1	Griffiths *et al*.^44^ (*m* < 1, shear-thinning)
11.	$\frac{2n}{(2m-1)n+1}$	0	Liao^64^
12.	1	0	Cortell^16^; Howell *et al*.^65^ (Heat transfer)

In this paper, an attempt has been made to study the non-Newtonian fluid effects on the two-dimensional boundary layer flow for different $\beta$ and $\varepsilon$. We note that $\varepsilon  > 1$ and $\varepsilon  < 0$ cases correspond respectively to wedge is moving faster and slower than mainstream velocity in opposite direction, while $\varepsilon \in (0.5,1.0)$ and $\varepsilon \in (0.0,0.5)$ cases are analogous as above but in the same direction. At this stage, it is also to note that for $n > 0$ and $n < 0$ cases correspond to accelerated and decelerated pressure gradient in the boundary-layer while $n=0$ is the Blasius flow of non-Newtonian fluid. The flow of Newtonian fluid ($m=1$) clearly demarcates the shear-thinning fluids ($m < 1$) from the shear-thickening fluids ($m > 1$). We employ the Chebyshev collocation method(CCM) for solution of the system (18) and (19) in which CCM effectively captures the fractional powers ($m-1$), the details of CCM are given below.

## Computational procedure

In recent years, the Chebyshev collocation methods are gaining wide popularity since the methods are robust and provide solutions which complements precisely the physical nature of the flow. While the CCM is well known for linear or mildly nonlinear fluid mechanics problems, the application of CCM to the problem in question is non-standard. We stress that the problems involving non-Newtonian fluid, we have not observed how to apply CCM. Thus, briefing the details of CCM is definitely of interest in its own right. We discuss the methodology below.

A standard ${i}^{th}$ order nonlinear ordinary differential equation can be written in the form

20 $$\begin{array}{c}\mathop{\sum }\limits_{i=0}^{I}\,\mathop{\sum }\limits_{k=0}^{K}\,{{\mathscr{P}}}_{i,k}{y}^{k}(\eta ){y}^{(i)}(\eta )+\mathop{\sum }\limits_{i=1}^{I}\,\mathop{\sum }\limits_{k=1}^{K}\,{{\mathscr{Q}}}_{i,k}{y}^{(i)}(\eta ){y}^{(k)}(\eta )\\ \,+\,\mathop{\sum }\limits_{i=1}^{I}\,\mathop{\sum }\limits_{k=1}^{K}\,{ {\mathcal R} }_{i,k}{({y}^{(i)}(\eta ))}^{p}{({y}^{(k)}(\eta ))}^{r}={\mathscr{V}}(\eta )\end{array}$$

where ${\mathscr{P}}$, ${\mathscr{Q}}$, ${\mathcal R}$ and ${\mathscr{V}}$ are known continuous functions of $\eta$ in $[a,b]$ and is supplied with necessary boundary conditions. With the help of a suitable linear transformation the interval $[a,b]$ can be transformed to $[\,-\,1,1]$ which is the domain of Chebyshev polynomials ${{\mathscr{T}}}_{n}(\eta )$ (Boyd^49^). Then $y(\eta )$ and its derivatives have a truncated Chebyshev series of the form

21 $$y(\eta )=\mathop{\sum }\limits_{n=0}^{{\mathscr{N}}}\,{a}_{n}{{\mathscr{T}}}_{n}(\eta )$$

and

22 $${y}^{(i)}(\eta )=\mathop{\sum }\limits_{n=0}^{{\mathscr{N}}}\,{a}_{n}^{(i)}{{\mathscr{T}}}_{n}(\eta )$$

where ${a}_{n}$ and ${a}_{n}^{(i)}$ are unknown Chebyshev coefficients of $y(\eta )$ and its derivatives. Let ${\eta }_{j}$ denote the Chebyshev collocation points defined as

$${\eta }_{j}=-\,\cos \,\left(\frac{j\pi }{{\mathscr{N}}}\right),\,j=0,1,2,\ldots ,{\mathscr{N}}$$

where ${\mathscr{N}}$ is a positive integer chosen at which the free stream condition is satisfied. The above Eqs. (21) and (22) have a matrix representation

23 $$y(\eta )={\mathscr{T}}(\eta )\,{\mathscr{A}}$$

with

24 $$y(\eta )=[y({\eta }_{0}),\,y({\eta }_{1}),\,\ldots ,\,y({\eta }_{{\mathscr{N}}}){]}^{{\prime} }$$

25 $${\mathscr{T}}(\eta )=[\begin{array}{cccc}{{\mathscr{T}}}_{0}({\eta }_{0}) & {{\mathscr{T}}}_{1}({\eta }_{0}) & \cdots  & {{\mathscr{T}}}_{{\mathscr{N}}}({\eta }_{0})\\ {{\mathscr{T}}}_{0}({\eta }_{1}) & {{\mathscr{T}}}_{1}({\eta }_{1}) & \cdots  & {{\mathscr{T}}}_{{\mathscr{N}}}({\eta }_{1})\\ \vdots  & \vdots  & \ddots  & \vdots \\ {{\mathscr{T}}}_{0}({\eta }_{{\mathscr{N}}}) & {{\mathscr{T}}}_{1}({\eta }_{{\mathscr{N}}}) & \cdots  & {{\mathscr{T}}}_{{\mathscr{N}}}({\eta }_{{\mathscr{N}}})\end{array}]$$

and

26 $${\mathscr{A}}=[\begin{array}{cccc}{a}_{0}, & {a}_{1}, & \ldots , & {a}_{{\mathscr{N}}}\end{array}]{\prime} $$

where ′ denotes a transpose of the quantities. Further, it readily follows that

27 $${y}^{(i)}(\eta )={\mathscr{T}}(\eta )\,{{\mathscr{A}}}^{(i)}.$$

Using the relation by coefficient matrix of $y(\eta )$ and its derivative (Sezer & Kaynak^50^; Dacscciouglu & Yaslan^46^)

28 $${{\mathscr{A}}}^{(i)}{\mathrm{=2}}^{i}{M}^{i}{\mathscr{A}}\mathrm{}.$$

Equation (27) can be written as

29 $${y}^{(i)}(\eta )={2}^{i}\,{\mathscr{T}}(\eta ){M}^{i}{\mathscr{A}}.$$

Similarly,

30 $${[y(\eta )]}^{k}={[\bar{y}(\eta )]}^{k-1}y(\eta )$$

where

$$\bar{y}=\bar{{\mathscr{T}}}\,\bar{{\mathscr{A}}},\,\bar{{\mathscr{T}}}=[\begin{array}{cccc}{\mathscr{T}}({\eta }_{0}) & 0 & \cdots  & 0\\ 0 & {\mathscr{T}}({\eta }_{1}) & \cdots  & 0\\ \vdots  & \vdots  & \ddots  & \vdots \\ 0 & 0 & \cdots  & {\mathscr{T}}({\eta }_{{\mathscr{N}}})\end{array}]\,{\rm{and}}\,diag(\bar{{\mathscr{A}}})={\mathscr{A}}$$

and these are evaluated at Chebyshev collocation points. Further simplifying using (29),

31 $${(\bar{y}(\eta ))}^{(k)}y{(\eta )}^{(i)}={2}^{k+i}\bar{{\mathscr{T}}}{(\bar{M})}^{k}\bar{{\mathscr{A}}}\,{\mathscr{T}}\,{M}^{i}{\mathscr{A}}$$

where $diag(\bar{M})=M$. Consequently, the matrix form of (20) can be written as

32 $$\begin{array}{c}\mathop{\sum }\limits_{i=0}^{I}\,\mathop{\sum }\limits_{k=0}^{K}\,{2}^{i}{{\mathscr{P}}}_{i,k}{(\bar{{\mathscr{T}}}\bar{{\mathscr{A}}})}^{k}{\mathscr{T}}\,{M}^{(i)}{\mathscr{A}}\\ \,+\,\mathop{\sum }\limits_{i=1}^{I}\,\mathop{\sum }\limits_{k=1}^{K}\,{2}^{(i+k)}{{\mathscr{Q}}}_{i,k}\bar{{\mathscr{T}}}\,{\bar{M}}^{k}\bar{{\mathscr{A}}}\,{\mathscr{T}}\,{M}^{i}A\\ \,+\,\mathop{\sum }\limits_{i=1}^{I}\,\mathop{\sum }\limits_{k=1}^{K}\,{2}^{(ip+lr)}{{\mathscr{R}}}_{i,k}{(\bar{{\mathscr{T}}}{\bar{M}}^{i}\bar{{\mathscr{A}}})}^{p}{({\mathscr{T}}{M}^{l}A)}^{r}={\mathscr{V}}\end{array}$$

where $$diag({{\mathscr{P}}}_{i,k})={{\mathscr{P}}}_{i,k}(\eta )$$, $$diag({{\mathscr{Q}}}_{i,k})={{\mathscr{Q}}}_{i,k}(\eta )$$, $$diag({{\mathscr{R}}}_{i,k})={{\mathscr{R}}}_{i,k}(\eta )$$, $${\mathscr{V}}=[{\mathscr{V}}({\eta }_{0}),{\mathscr{V}}({\eta }_{1}),\ldots ,{\mathscr{V}}({\eta }_{{\mathscr{N}}})]{\prime} $$.

More concisely (32) takes the form

33 $$W{\mathscr{A}}=V\mathrm{}.$$

The above equation represents a system of $({\mathscr{N}}+\mathrm{1)}$ nonlinear algebraic equations involving unknown Chebyshev coefficients ${\mathscr{A}}$ given by (26). Further, incorporating the boundary conditions, we obtain the modified augmented matrix

34 $$\tilde{W}{\mathscr{A}}=\tilde{V}$$

which on inversion gives (26), the required solution can be computed from (21). On the other hand, to solve the flow equation of the model considered here (18) and (19), we first transform the semi-infinite flow domain $[0,\infty )$ to $[\,-\,1,1]$ by using the following transformations

35 $$\phi (\eta )=\frac{{\eta }_{\infty }}{2}\phi (\zeta )\,{\rm{where}}\,\zeta =\frac{2\eta }{{\eta }_{\infty }}-1$$

where ${\eta }_{\infty }$ is a large infinity value at which the boundary condition is met. Substitution of  (35) in (18) and (19) gives nonlinear ordinary differential equation in the interval $[\,-\,1,1]$ for which the solution can be written in terms of the truncated Chebyshev series as

36 $$\phi (\zeta )=\mathop{\sum }\limits_{n=0}^{N}\,{x}_{n}^{(i)}{{\mathscr{T}}}_{n}(\zeta )\,{\rm{or}}\,F={\mathscr{T}}\,X$$

where *X* is the unknown coefficient vector of the Chebyshev polynomials. Following (22), (29), (30) and (31), we have the final matrix form as

37 $$\begin{array}{c}[m|{2}^{2}\,\tilde{{\mathscr{T}}}\,{\bar{M}}^{2}\bar{X}{|}^{m-1}({2}^{3}\,{\mathscr{T}}\,{M}^{3})+\frac{2}{m+1}a\bar{{\mathscr{T}}}\,\bar{X}({2}^{2}\,{\mathscr{T}}\,{M}^{2})\\ \,-\,a\beta ({2}^{2}\,\bar{{\mathscr{T}}}\,\bar{M}\bar{X}\,{\mathscr{T}}\,M)]X=-\,a\beta {\varepsilon }^{2} {\mathcal I} \end{array}$$

where ${\mathcal{I}}$ is a unit vector of order $({\mathscr{N}}+1,1)$. The challenges one confronts to solve (18) are the nonlinearity of the equation and further computation of $|{2}^{2}\,\bar{{\mathscr{T}}}\,{\bar{M}}^{2}\bar{X}{|}^{m-1}$ when $m\ne 1$. We can overcome the latter concern by performing simple computational linear algebra. A close examination reveals that the matrix which is obtained as a product form of ${2}^{2}\,\bar{{\mathscr{T}}}\,{\bar{M}}^{2}\bar{X}$ is diagonalizable. Hence, it can be written in terms of its eigenvalues $D$ and eigenvectors $C$ as

$$|{2}^{2}\,\bar{{\mathscr{T}}}\,{\bar{M}}^{2}\bar{X}{|}^{m-1}{\mathrm{=2}}^{2}|{\mathscr{C}}\,{{\mathscr{D}}}^{m-1}\,{{\mathscr{C}}}^{-1}|$$

in all the simulations and our scientific code essentially performs the diagonalization. Hence we can write the augmented matrix as

38 $$WX=S$$

where

$$\begin{array}{rcl}W & = & [m\,{2}^{2}|{\mathscr{C}}\,{{\mathscr{D}}}^{m-1}\,{{\mathscr{C}}}^{-1}|({2}^{3}\,{\mathscr{T}}\,{M}^{3})+\frac{2}{m+1}a\bar{{\mathscr{T}}}\,\bar{X}({2}^{2}\,{\mathscr{T}}\,{M}^{2})\\  &  & -\,a\beta ({2}^{2}\,\bar{{\mathscr{T}}}\,\bar{M}\bar{X}\,{\mathscr{T}}\,M)]\end{array}$$

and

$$S=-\,a\beta {\varepsilon }^{2}\, {\mathcal I} \,{\rm{and}}\,a={\left(\frac{{\eta }_{\infty }}{2}\right)}^{(m+1)}.$$

We now incorporate the boundary conditions (19) by using (29).

39 $${\mathscr{T}}(\,-\,1)X=0,\,2{\mathscr{T}}(\,-\,1)MX=1-\varepsilon \,{\rm{and}}\,2{\mathscr{T}}(1)MX=\varepsilon .$$

We modify Eq. (38) by plugging the row matrices (39) at first, second and last row of (38) respectively. Therefore, we obtain the modified augmented matrix of the form

40 $$\tilde{W}X=\tilde{S}.$$

**Figure 2:**
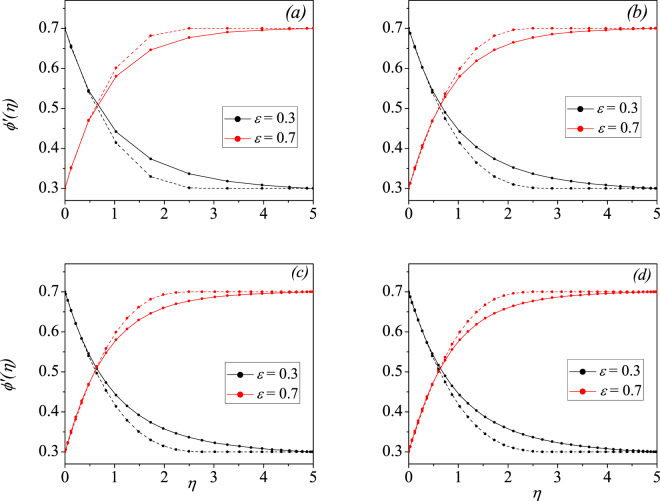
Velocity profiles of shear thinning (*m* = 0.6, solid) and shear thickening (*m* = 1.4, dashed) fluid, at $n=0.5$ for different ${\mathscr{N}}$ number of terms considered (**a**) ${\mathscr{N}}$ = 10, (**b**) ${\mathscr{N}}$ = 20, (**c**) ${\mathscr{N}}$ = 30, (**d**) ${\mathscr{N}}$ = 40.

Since *X* is unknown, the left hand side of (37) represents the augmented form of non-linear algebraic equations and this can be handled by taking an initial approximation to $X$ (in the present computation $X$ is taken as a zero vector of order $({\mathscr{N}}+1,1)$ chosen at the first phase of computation) and updating it at each stage of calculation for sufficient ${\mathscr{N}}$ until a desired tolerance of ${10}^{-6}$ is attained. We observed that, the number of terms required to achieve the desired results were different for different $m$. Suppose $m=1$ we required a maximum of $30$ terms and for $m < 1$ say $m=0.6,0.8$ an approximate of $27$ terms were sufficient to get the desired accuracy whereas for $m > 1$ say $m=1.2,1.4$ number of terms required were $40$ (refer to Fig. 2 and Table 2 for details). All the results provided below are tested for their convergence criteria. Solving Eq. (40) for $X$, and hence retrieving to original domain by using (35) in (36) we can further compute the derivatives of (36) to obtain the velocity profiles and skin-friction coefficient associated with the Ostwald-de Waele flow. Figure 2 also emphasizes the convergence of the CCM as the computations are progressed with increasing the number of terms. At Fig. 2a, the curve is not sufficiently smooth, although conditions are clearly satisfied, but Fig. 2d simulates the required smooth profile. Table 2 also gives the corresponding wall shear stress values at each ${\mathscr{N}}$ values. From the table, it is observed that for $m=0.6$, the convergence occurs much before $40$ terms in the CCM, but to make table consistent it is given upto 40. On the other hand, the CCM is also tested by fixing the number of terms equal to $50$ and decreasing the tolerance value to ${10}^{-8}$. The results are indistinguishable in both cases, hence, we continued all our simulations with the former analysis.

**Table 2:** Convergent wall-shear stress values $\phi {\prime\prime} (0)$ obtained by Chebyshev collocation method for different ${\mathscr{N}}$.

${\mathscr{N}}$	*m* = 0.6		*m* = 1.4	
	*ε* = 0.3	*ε* = 0.7	*ε* = 0.3	*ε* = 0.7
10	−0.365911	0.387784	−0.396898	0.488379
20	−0.364826	0.388986	−0.416139	0.425052
30	−0.364826	0.388986	−0.416092	0.425495
40	−0.364826	0.388986	−0.416092	0.425495

## Large *η* asymptotics

In order to validate the presented numerical results achieved using CCM, we also perform asymptotic analysis for the system (18) and (19) when $\eta \gg 1$ with the objective of having an exact solution in terms of confluent hypergeometric functions. We follow the similar procedure given by (Sachdev *et al*.^4^; Kudenatti *et al*.^5^) for Newtonian fluid $m=1$ that is $|\phi {\prime} (\eta )-\varepsilon |\to 0$ as $\eta \to \infty$ which gives the form

41 $$\phi (\eta )\approx \varepsilon \eta +{\eta }_{0}+\int \,{\mathscr{S}}(\eta )d\eta $$

where ${\eta }_{0}$ is an arbitrary constant, ${\mathscr{S}}(\eta )$ and its derivatives are assumed small. Note that ${\eta }_{0}=0$ from (19). The above form (41) is valid for all $m$, $n$ and $\varepsilon$ and ${\mathscr{S}}(\eta )$ tends to zero away from the wedge surface. Substitution of (41) in (18) and (19) and upon linearizing, we have

42 $${\mathscr{S}}{\prime\prime} (\eta \rangle +\frac{2\varepsilon }{m(m+1\rangle }\eta {\mathscr{S}}{\prime} (\eta \rangle -\frac{4n\varepsilon }{m(1+(2m-1\rangle n\rangle }{\mathscr{S}}(\eta \rangle =0$$

with the conditions

43 $${\mathscr{S}}(0)=1-2\varepsilon ,\,{\mathscr{S}}(\infty )=0.$$

Using the transformations

44 $${\mathscr{S}}(\eta )=\hat{{\mathscr{S}}}(\hat{\eta }),\,\hat{\eta }=\frac{\varepsilon {\eta }^{2}}{m(m+1)}$$

in (42), we get

45 $$\hat{\eta }\hat{{\mathscr{S}}}{\prime\prime} (\hat{\eta })+\left(\frac{1}{2}+\hat{\eta }\right)\hat{{\mathscr{S}}}{\prime} (\hat{\eta })-\beta \left(\frac{n+1}{2}\right)\hat{{\mathscr{S}}}(\hat{\eta })=0$$

which has a solution

46 $$\begin{array}{rcl}\hat{{\mathscr{S}}}(\hat{\eta }) & = & {e}^{-\hat{\eta }}\sqrt{\hat{\eta }}[{c}_{1}{\mathscr{U}}\left(\frac{1}{2}(2+(m+1)\beta );\frac{3}{2};\hat{\eta }\right)\\  &  & +\,{c}_{2} {\mathcal L} \left(-\frac{1}{2}(2+(m+1)\beta );\frac{1}{2};\hat{\eta }\right)]\end{array}$$

where $\beta =\frac{2n}{1+(2m-1)n}$, ${\mathscr{U}}(\cdot ,\cdot ,\cdot )$ and ${\mathscr{L}}(\cdot ,\cdot ,\cdot )$ are the confluent hypergeometric function of second kind and Laguerre function respectively, and ${c}_{1}$ and ${c}_{2}$ are arbitrary constants (Abramowitz & Stegun^51^ and Andrews^52^). The special functions ${\mathscr{U}}$ and ${\mathscr{L}}$ can be further transformed into the confluent hypergeometric function of first kind using

47a $$\begin{array}{rcl}{\mathscr{U}}(\hat{a},\hat{b},\hat{z}) & = & \frac{\pi }{\sin (\hat{b}\pi )}[\frac{{\mathscr{M}}(\hat{a},\hat{b},\hat{z})}{\Gamma (1+\hat{a}-\hat{b})\Gamma (\hat{b})}\\  &  & -\,{\hat{z}}^{1-\hat{b}}\frac{{\mathscr{M}}(1+\hat{a}-\hat{b},2-\hat{b},\hat{z})}{\Gamma (2-\hat{b})\Gamma (\hat{a})}],\end{array}$$

47b $$ {\mathcal L} (\hat{a},\hat{b},\hat{z})=\frac{\Gamma (\hat{a}+\hat{b}+1)}{\Gamma (1+\hat{a})\,\Gamma (1+\hat{b})}{\mathscr{M}}(\,-\,\hat{a},1+\hat{b},\hat{z}).$$

Therefore, utilizing (47), (46) can be rewritten in original variables (44) as

48 $$\begin{array}{rcl} {\mathcal L} (\eta ) & = & {e}^{-\frac{\varepsilon {\eta }^{2}}{m(m+1)}}\sqrt{\frac{\varepsilon {\eta }^{2}}{m(m+1)}}\,[-\sqrt{\pi }\,{c}_{1}(2\frac{{\mathscr{M}}\left(1+\Delta ;\frac{3}{2};\frac{\varepsilon {\eta }^{2}}{m(m+1)}\right)}{\Gamma \left(\frac{1}{2}+\Delta \right)}\\  &  & -\,\sqrt{\frac{m(m+1)}{\varepsilon {\eta }^{2}}}\frac{{\mathscr{M}}\left(\frac{1}{2}+\Delta ;\frac{1}{2};\frac{\varepsilon {\eta }^{2}}{m(m+1)}\right)}{\Gamma (1+\Delta )})\end{array}$$

49 $$\begin{array}{lll} &  & +\,{c}_{2}\frac{2}{\sqrt{\pi }}\frac{\Gamma \left(\frac{1}{2}-\Delta \right)}{\Gamma (\,-\,\Delta )}\,{\mathscr{M}}\left(1+\Delta ;\frac{3}{2};\frac{\varepsilon {\eta }^{2}}{m(m+1)}\right)].\end{array}$$

where $\Delta =\frac{(m+1)}{2}$. Employing the first condition ${\mathscr{S}}(0)=1-2\varepsilon$ in (48) gives

50 $${c}_{1}=\frac{1}{\sqrt{\pi }}(1-2\varepsilon )\,\Gamma (1+\Delta ).$$

To eliminate ${c}_{2}$, we utilize another relation

51 $${\mathscr{M}}(\hat{a},\hat{b},\hat{z})=\frac{{e}^{\pm i\pi \hat{a}}{\hat{z}}^{-\hat{a}}\Gamma (\hat{b})}{\Gamma (\hat{b}-\hat{a})}+\frac{{e}^{\hat{z}}{\hat{z}}^{\hat{a}-\hat{b}}\Gamma (\hat{b})}{\Gamma (\hat{a})}.$$

In view of (51) for $\hat{\eta }=\frac{\varepsilon {\eta }^{2}}{m(m+1)}\to \infty$ as $\eta \to \infty$ in (48) gives ${c}_{2}=0$. Thus, the complete solution is

52 $$\begin{array}{rcl}{\mathscr{S}}(\eta ) & = & 2(2\varepsilon -1)\sqrt{\frac{\varepsilon {\eta }^{2}}{m(m+1)}}\frac{\Gamma \left(\frac{2+(m+1)\beta }{2}\right)}{\Gamma \left(\frac{1+(m+1)\beta }{2}\right)}{e}^{-\frac{\varepsilon {\eta }^{2}}{m(m+1)}}\\  &  & \times \, {\mathcal M} \left(\frac{2+(m+1)\beta }{2};\frac{3}{2};\frac{\varepsilon {\eta }^{2}}{m(m+1)}\right)\\  &  & +\,(1-2\varepsilon )\,{e}^{-\frac{\varepsilon {\eta }^{2}}{m(m+1)}}\, {\mathcal M} \left(\frac{1+(m+1)\beta }{2};\frac{1}{2};\frac{\varepsilon {\eta }^{2}}{m(m+1)}\right).\end{array}$$

Employing Kummer’s transformation, we arrive at

53 $$\begin{array}{rcl}\phi {\prime} (\eta ) & = & \varepsilon +2(2\varepsilon -1)\sqrt{\frac{\varepsilon {\eta }^{2}}{m(m+1)}}\frac{\Gamma \left(\frac{2+(m+1)\beta }{2}\right)}{\Gamma \left(\frac{1+(m+1)\beta }{2}\right)}\\  &  & \times \,{\mathscr{M}}\left(\frac{1-(m+1)\beta }{2};\frac{3}{2};-\frac{\varepsilon {\eta }^{2}}{m(m+1)}\right)\\  &  & +\,(1-2\varepsilon )\,{\mathscr{M}}\left(\frac{\,-\,(m+1)\beta }{2};\frac{1}{2};-\,\frac{\varepsilon {\eta }^{2}}{m(m+1)}\right).\end{array}$$

**Figure 3:**
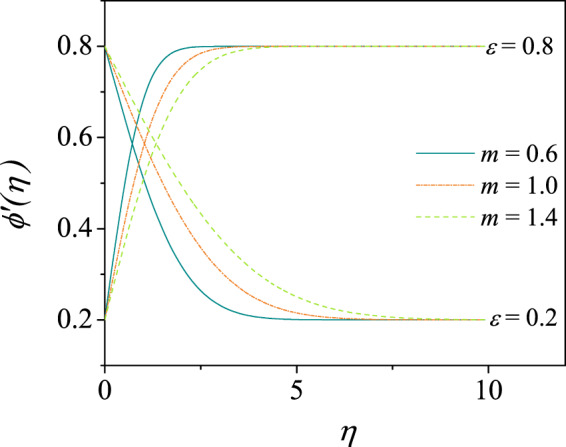
Velocity profiles for the shear-thinning ($m=0.6$) and shear-thickening ($m=1.4$) boundary layer flow over a moving wedge(or stretching sheet) for $\varepsilon =0.2$ and $\varepsilon =0.8$ with $n=0.5$ demarcated by velocity profiles for the Newtonian fluid flow ($m=1.0$). These results are obtained by asymptotic solution (53).

For $m < 1$ (shear-thinning fluids) Denier & Dabrowski^45^ have derived the asymptotic expression

54 $$\phi {\prime} (\eta )=1-{\left(\frac{1-m}{2m}\right)}^{\frac{m}{m-1}}\left(\frac{2m}{m+1}\right){\eta }^{\frac{m+1}{m-1}}+\cdots $$

for zero-pressure gradient $n=0$ and $\varepsilon =1$. This form certainly predicts the far-field behavior, but with larger domain. Nevertheless, the solution given by (53) is valid for all $m$, $n$ and $\varepsilon$ and effectively captures (54) as a special case. The solution for ${\mathscr{S}}(\eta )$ and hence the velocity profile is given by (53). Some of the velocity profiles $\phi {\prime} (\eta )$ obtained from (53) are shown in Fig. 3 for different $\varepsilon$ and $m$ values.

From Fig. 3, it is noticed that, all the velocity shapes are decaying to the mainstream flow asymptotically. This validates the assumption (41) that ${\mathscr{S}}(\eta )$ in (52) tends to zero as $\eta \to \infty$. The solution (53) takes smaller domain to satisfy the end condition which assures the exponential-type decay of the solutions. The expression (54) given by (Denier & Dabrowski^45^) is valid for $m < 1$ and decays to the mainstream algebraically. However, the solution (53) is valid even for $m > 1$, $m=1$, $m < 1$ as is clearly seen in Fig. 3 and for any $\varepsilon$. For $\varepsilon =1$, it is further observed that the boundary layer thickness is found to be decreasing for increasing $m$ value. This is in agreement with those given by Agarwal *et al*.^53^ who have studied the boundary layer flow of non-Newtonian fluid over a thin needle.

## Numerical results and discussion

The velocity ratio parameter $$\varepsilon $$$$(\,=\,\frac{{U}_{\infty }}{{U}_{\infty }+{U}_{0}})$$ mathematically combines two different boundary layer problems. Two extreme cases $$\varepsilon =0$$ and $$\varepsilon =1$$ correspond to boundary layer flow due to stretching surface and over a constant wedge. For other values of $$\varepsilon $$, boundary-layer flow over a moving wedge admits a class of self-similar solutions even for non-Newtonian fluid. When $$\varepsilon  > 1$$ and $$\varepsilon  < 0$$, the mainstream flow has a slower and faster velocity than the wedge and both are moving in opposite directions. However, when $$\varepsilon  < 1$$ and $$\varepsilon  > 0$$, both wedge and mainstream move in the same direction. While for $$\varepsilon =0.5$$, wedge and mainstream have the same speed in the same direction and hence produce an exact solution $$\phi (\eta )=\frac{\eta }{2}$$ for all $$\beta $$ and $$m$$, at which the wall shear stress vanishes and hence demarcates the solution structure. Thus, the velocity ratio $$\varepsilon $$ signifies the general boundary layer flows possessing the horizontal velocity profiles that decay to the mainstream flows asymptotically. The present analysis is fully rational and unique which sheds some light on the interesting flow phenomena. Kudenatti^32^ tackles an equally valid problem in the range $$\varepsilon \in (0,0.5)$$ for $$m=1$$(Newtonian case). Rather than studying the problem numerically, he gave an analytical solution for all $$\beta $$.

Further, these numerical simulations obtained by CCM can be matched comparatively with results obtained by Kudenatti^32^ in case of Newtonian fluid ($m=1$ in (18)). Table 3 compares various results obtained by the CCM with the analytical solution given by Kudenatti^32^ in terms of the wall shear stress $\phi {\prime\prime} \mathrm{(0)}$ for various $\varepsilon$, $\beta$ and for $m=1$ (Newtonian case). An iterative scheme in CCM is used in which the velocity shapes and the wall shear stress are determined i.e, the number of terms required to produce these results is then adjusted in response to the error-tolerance fixed for the simulation. The results produced by the CCM agreed well with those given via analytical solutions in the range of $\varepsilon \in (0,0.5)$. It is further noticed that the accuracy is found to be better for increasing $\varepsilon$ and $\beta$. The $\phi {\prime\prime} \mathrm{(0)}$ value becomes zero and changes its sign at $\varepsilon =0.5$ for all $\beta$ and $m$. We also note that the convergent solution $\phi {\prime\prime} \mathrm{(0)}$ obtained for certain parameters is used as an initial condition for consistency in the simulations. Table 3 also ensures that there exists a velocity profile for each value which is benign and satisfies the boundary conditions asymptotically.

**Table 3:** Comparison of wall-shear stress values $\phi {\prime\prime} (0)$ obtained by Chebyshev collocation method for various stretching rate parameter *ε* at pressure-gradient parameters *β* for *m* = 1 with those declared by Kudenatti^32^.

*β*	*ε*	Kudenatti^32^	CCM
0.0	0.1	−0.492625	−0.494003
	0.2	−0.363901	−0.363417
	0.3	−0.237219	−0.237157
	0.4	−0.115811	−0.115815
	0.5	0.0	0.0
0.5	0.1	−0.675918	−0.675010
	0.2	−0.513980	−0.513392
	0.3	−0.346194	−0.346623
	0.4	−0.175403	−0.175339
	0.5	0.0	0.0
1.0	0.1	−0.823981	−0.823674
	0.2	−0.633671	−0.633833
	0.3	−0.432951	−0.432617
	0.4	−0.221982	−0.221091
	0.5	0.0	0.0

**Figure 4:**
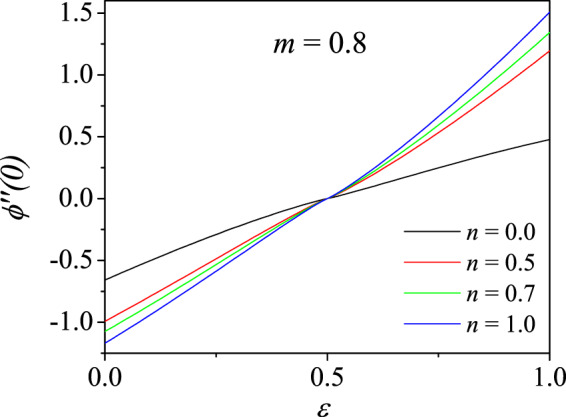
Variation of wall shear stress $\phi {\prime\prime} \mathrm{(0)}$ with velocity ratio parameter $\varepsilon$ for different pressure gradient parameters *n* for shear-thinning fluid ($m=0.8$).

**Figure 5:**
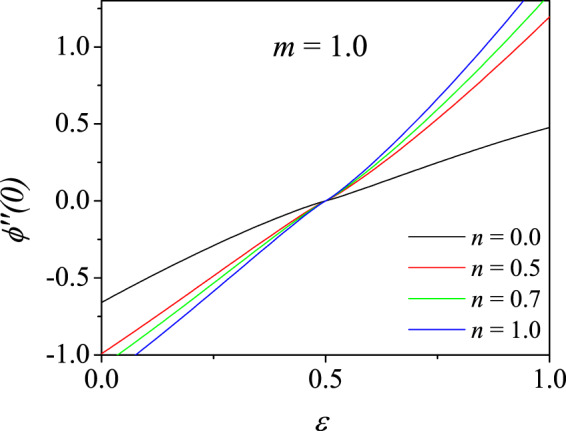
Variation of wall shear stress $\phi {\prime\prime} \mathrm{(0)}$ with velocity ratio parameter $\varepsilon$ for different pressure gradient parameters *n* for Newtonian fluid ($m=1.0$).

**Figure 6:**
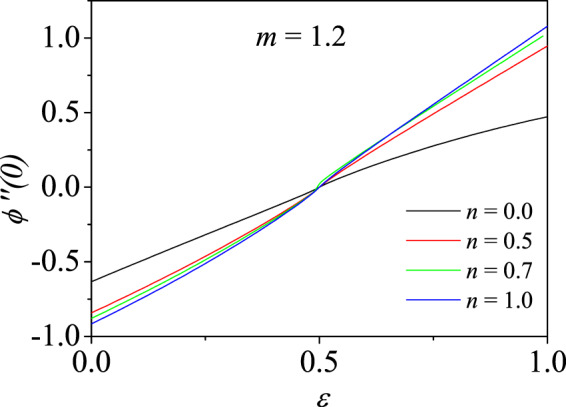
Variation of wall shear stress $\phi {\prime\prime} \mathrm{(0)}$ with velocity ratio parameter $\varepsilon$ for different pressure gradient parameters *n* for shear thickening fluid ($m=1.2$).

We now study the broader structure of the boundary layer flow over a moving wedge for various velocity ratio parameter $\varepsilon$ and non-Newtonian index parameter $m$ and are given in terms of the wall shear stress $\phi {\prime\prime} \mathrm{(0)}$. These results are just analogous to those presented in Table 3 for $m=1$. The various $\phi {\prime\prime} \mathrm{(0)}$ for different $m$, pressure gradient $n$, and $\varepsilon \in [0,1]$ are given in Figs. 4, 5 and 6. These results are obtained by the convergent CCM. It is immediately clear that the wall shear stress $\phi {\prime\prime} \mathrm{(0)}$ identically vanishes at $\varepsilon =0.5$ for all $n$ and $m$, thereby validating the trivial exact solution $\phi (\eta )=\varepsilon \eta$. All $\phi {\prime\prime} \mathrm{(0)}$ values are negative for $\varepsilon \in [0,0.5)$ and became positive in the range $\varepsilon \in (0.5,1]$.

In other words, for increasing $\varepsilon$ from 0, $\phi {\prime\prime} \mathrm{(0)}$ also increases. When $\varepsilon  < \frac{1}{2}$, the wall shear stress values are higher for smaller $n$ while for $\varepsilon \ge \frac{1}{2}$, the trend is reversed which is observed clearly in the figure. The similar trend is observed for all $m$ and $n$. Karkera *et al*.^33^ have shown, for Newtonian fluid, similar solution structure including the double solutions when the pressure gradient parameter $n$ is negative.

**Figure 7:**
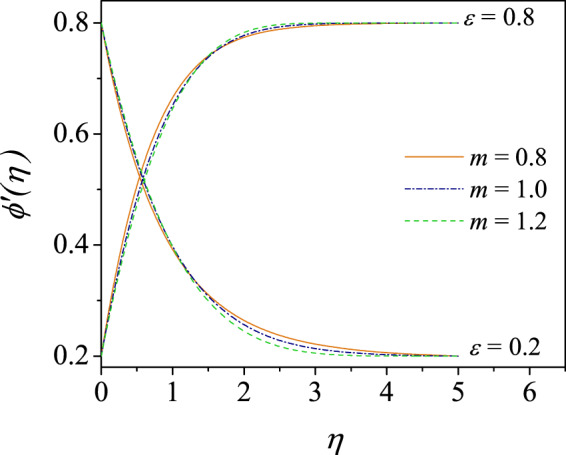
Velocity profiles for $\varepsilon =0.2$ and $\varepsilon =0.8$ for shear-thinning ($m=0.8$, solid), Newtonian ($m=1$, dash-dotted) and shear-thickening ($m=1.2$, dashed) power-law fluid for pressure gradient parameter $n=1.0$.

In order to realize the velocity profiles in the two-dimensional boundary layer in the power-law fluids, we have plotted the velocity shapes $\phi {\prime} (\eta )$ in Fig. 7 as a function of $\eta$ for $\varepsilon =0.2$ and $\varepsilon =0.8$ and for shear-thinning and shear-thickening fluids. It is clearly seen that the velocity profiles satisfy the boundary conditions asymptotically as $\eta \to \infty$. These shapes further confirm that the shear-thinning fluid $m < 1$ makes the boundary layer thickness thinner compared to the shear-thickening fluid $m > 1$. These two cases are clearly separated by the Newtonian fluid $m=1$. The results are further confirmed by the asymptotic solution (53) that are given in Fig. 3 although for different value $\varepsilon$ values.

**Figure 8:**
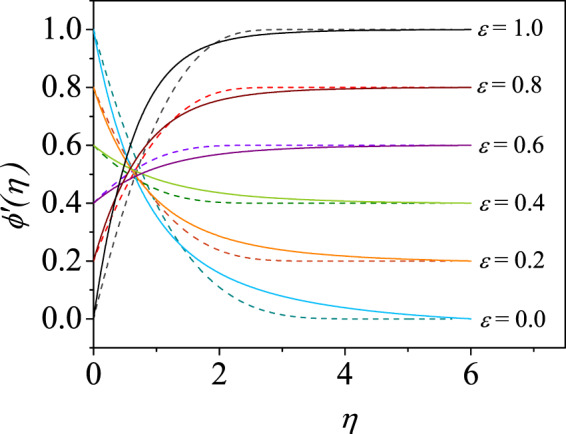
Velocity profiles for some representative values of $\varepsilon \in [0,1]$ for shear-thinning ($m=0.6$, solid) and shear-thickening ($m=1.4$, dashed) power-law fluid for pressure gradient parameter $n=0.5$.

Figure 8 depicts the velocity profiles obtained by the CCM for different $\varepsilon \in [0,1]$ including two extreme cases $\varepsilon =0$ and $\varepsilon =1$ for shear-thinning and shear-thickening fluids. These shapes have been simulated using the CCM. The quality rheological interpretation of the solution for different $\varepsilon$ tested and for shear thinning fluid deviates slightly from the shear-thickening fluid, which is clearly seen in Fig. 8. Around $\varepsilon =0.5$, the curves criss-cross at $\eta \sim 0.5$ and the criss-cross is shifting towards the wedge surface for shear-thickening fluids.

Yurusoy^54^ has shown for $\varepsilon =0$ that exactly similar velocity shapes for shear-thinning and shear-thickening fluids wherein the velocity profiles approach the mainstream flow exponentially for $m\le 1$ and for $m > 1$, the shapes take larger distance to approach the mainstream. However, the CCM effectively captures these characteristics of the profiles in a smaller domain as seen in the figure. For two values of $\varepsilon$, some of the fluid velocity curves have been reproduced in Fig. 7 when $m$ is varied to study the Ostwald-de Waele fluid effects on the boundary layer flow. These results are analogous to those produced in Fig. 3 which are for $m=0.6,\,1,\,1.4$. The solutions for specific value of $\varepsilon ,$ say 0.2, the curves for $m=0.8$ and $m=1.2$ crossing the Newtonian case $m=1$ at some $\eta \simeq 0.7$ and the similar structure appears for $\varepsilon =0.8$ which is seen in the figure. However, the asymptotic solutions are given by (53) in Fig. 3 have no cross-over, this may be attributed to the exact solutions given in terms of the confluent hypergeometric functions.

**Figure 9:**
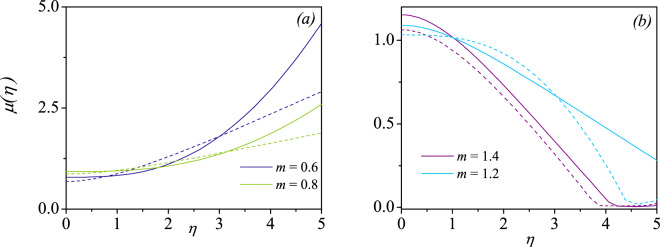
Viscosity profiles $\mu (\eta )=m|\phi {\prime\prime} \mathrm{(0)}{|}^{m-1}$ versus $\eta$ for $\varepsilon =0$ (solid line) and $\varepsilon =1$ (dashed)line for (**a**) shear-thinning fluids and (**b**) shear-thickening fluids.

Figure 9 represents the viscosity profiles for both shear-thinning and shear-thickening fluids. The viscosity modification of a generalized Ostwald-de Waele fluid is defined as $\mu (\eta )=m|\phi {\prime\prime} (\eta ){|}^{m-1}$. For a Newtonian fluid, i.e, for $m=1$, $\mu =1$, a constant viscosity is predicted which is obvious. Two representative values of $\varepsilon$, say $\varepsilon =0$ and $\varepsilon =1$ are chosen and for a favorable pressure gradient parameter $n=1$. From Fig. 9, it can be readily observed that, for the shear-thinning fluids ($m=0.6$ and $m=0.8$), there is quite large viscosity within the confinement of the boundary layer region. This is considered to be unphysical for the power-law fluids. An enhancement in viscosity is still larger for $\varepsilon =0$ compared to $\varepsilon =1$ case. On the other hand, for shear-thickening fluids ($m=1.2$ and $m=1.4$), a finite viscosity is predicted which approaches to zero as $\eta \to \infty$. This clearly shows the viscosity effects are completely neglected when an inviscid flow is approached. We noticed, although not shown here, that for the increasing pressure gradient, the viscosity on the surface ($\mu \mathrm{(0)}$) is found to be decreasing for shear-thinning fluids. It is also noticed that the viscosity increases unboundedly away from the wedge surface as the pressure gradient $\beta$ increases. While the nature of $\mu \mathrm{(0)}$ and the viscosity has a reverse trend for the shear-thickening fluids.

## Conclusions

The present work deals with the hydrodynamic flow a non-Newtonian power-law fluid (Ostwald-de Waele fluid) model past a moving wedge or a stretching sheet in a unified manner. The wedge/sheet is assumed to move/stretched with a velocity varying as the power of the distance from the leading edge of the boundary-layer along with the freestream. With the help of suitable similarity transformations, the governing partial differential equation system was then reduced to the non-linear ordinary differential equation and then solved numerically by Chebyshev collocation technique. The application of the collocation method for the problem under consideration is first of its kind hence we propose an asymptotic solution which is in validation with these numerical results. The model was discussed for the shear-thinning and shear-thickening power-law fluids, and a comparison was made with the Newtonian solutions. Results for the skin friction coefficient, velocity profiles as well as viscosity profiles are presented for different values of the governing parameters. It is noticed that the wall-shear stress increases with an increase in $\varepsilon$, the velocity ratio parameter and decrease in the power-law index *m*. Further, the boundary-layer thickness for shear-thinning fluids is small in comparison with that of shear-thickening power-law fluids for a given set of parameters. Physically, the flow for $m < 1$ is always adhered to the wedge surface thereby making the flow benign whereas for $m > 1$, the flow is convected towards the mainstream. It is also shown that with the increase in shearing stresses, the viscosity of the shear-thinning power-law fluids $m < 1$ increases unboundedly in the confinement of the boundary layer whereas it is always finite and approaches zero as $\eta \to \infty$ for shear-thickening fluids.
